# Writing the History of Endemic Viral Disease: The Case of Bovine Viral Diarrhoea, c.1945–1980

**DOI:** 10.1093/shm/hkab131

**Published:** 2022-02-11

**Authors:** Abigail Woods

**Keywords:** endemic disease, bovine viral diarrhoea, livestock, veterinary medicine, virology

## Abstract

In Western countries during the post-World War II decades, endemic viral diseases were increasingly important to health. Such diseases have attracted limited historical attention. Due to changing methods of livestock production, they were particularly prevalent on the farm. This article uses a case study of the cattle disease, bovine viral diarrhoea (BVD), to demonstrate their historical significance. Spanning North America, the UK and Australia, it reveals the complex nature of BVD, and how and why its clinical, aetiological, epidemiological and host species identities evolved over time. This analysis sheds new light on how endemic viral diseases of livestock were experienced, understood and distributed in this period, and the influence exerted by changing agricultural practices, concerns about biological warfare and the development of virology as applied to veterinary medicine.

If Covid-19 had emerged as an everyday endemic respiratory disease rather than the cause of a global pandemic, would future scholars show any interest in its history? Existing scholarship suggests not. It is over 50 years since virologists created the coronavirus category to which Covid-19 now belongs, and began to identify the viruses they located in it as causes of diseases like the common cold in humans, epidemic diarrhoea in pigs, infectious bronchitis in poultry and infectious peritonitis in cats.^1^ However, coronaviruses did not attract attention beyond scientific circles until the twenty-first century, when they were found responsible for Severe Acute Respiratory Syndrome (SARS, first reported 2002) and Middle East Respiratory Syndrome (MERS, first reported in 2012). This interest was stimulated by the novelty of SARS and MERS, their rapid transmission, the human death rate and the discovery that the viruses responsible had originated in animals. Due to these features, their histories have already been written,^2^ while other longer-standing diseases caused by coronaviruses remain in historical obscurity.^3^

This example illustrates two converging medical historiographical trends: the privileging of epidemic over endemic diseases, and of human diseases—and those spreading to humans from animals—over those affecting animals alone. These trends have skewed historical understandings of disease in Western countries during the post-World War II (WWII) decades, to the extent that virtually nothing is known about the history of the infectious diseases to which vertebrate species were most frequently subject. This situation can be attributed partly to medical history’s anthropocentricity, which endures in spite of growing historical, sociological and environmental evidence of the mutual shaping of human and animal lives.^4^ It also reflects the enduring appeal of epidemics as historically revealing subjects that are relatively easy to investigate. As Charles Rosenberg famously observed, epidemics ‘constitute an extraordinarily useful sampling device—at once found objects and natural experiments capable of illuminating fundamental patterns of social value and institutional practice’.^5^ As ‘an event, not a trend’,^6^ they generate copious, chronologically focussed responses, whose documentation offers a straightforward entry point for the historian.

Like MERS and SARS, many of the epidemics that historians have examined in Western countries during the post-WWII era were caused by viruses. As disease agents, their relative importance was growing as rising living standards, public health interventions, and pharmaceuticals like vaccines and antibiotics reduced the impact of prevalent bacterial diseases. Understandings of their nature and effects were also expanding as a consequence of what Gaudillière and Creager refer to as new ‘experimental arrangements’—a combination of tools, techniques and operators that increased virus visibility and manipulability, and contributed to the emergence of virology as a specialist field of enquiry.^7^ The evolution of viral epidemic disease and linked developments in virological science have been examined in histories of epidemics of influenza and AIDS (both of which originated in animals),^8^ and for viral diseases like polio that reached epidemic proportions in children.^9^ They also feature in accounts of the animal viral epidemics of foot and mouth disease, myxomatosis and rinderpest.^10^ However, little is known about encounters in the field and the laboratory with contemporaneous endemic viral diseases.

By definition, these diseases were always present in certain populations and regions. Typically affecting the respiratory or gastro-intestinal systems, in Western countries they were rarely fatal, but had a significant, cumulative impact on health, well-being and productive capacity.^11^ Many had complex aetiologies that challenged existing understandings of the relationship between infection and disease.^12^ In the decades after WWII they were particularly prevalent in farmed livestock due to the adoption of more intensive livestock husbandry systems. Promoted by agricultural policies, and investments in science and technology that aimed to boost food output in the wake of wartime food shortages, these systems unexpectedly encouraged viral emergence and spread, with detrimental implications for livestock health.^13^ Such circumstances provide a compelling justification for why efforts to overcome medical historians’ traditional neglect of endemic and non-human diseases should commence with the study of endemic viral diseases of farmed livestock.

Investigating such diseases poses certain methodological challenges. They were rarely targeted by state-led controls or public education campaigns. Archival material is therefore sparse, forcing reliance on published scientific literature that is often extremely technical. While the understandings and identities of most diseases changed over time, there is something particularly unstable and slippery about the loosely defined and variously presenting viral diseases that affected Western livestock in the post-WWII decades. Nevertheless, examining how actors dealt with this complexity can be historically productive, as historians have demonstrated for other indeterminate diseases.^14^

This article will begin the process of recording and analysing the historical significance of endemic viral diseases of animals through a case study of bovine viral diarrhoea (BVD). Although barely known outside of farming circles, BVD is regarded today as an extremely important cause of immunosuppression, reproductive problems and reduced milk yield in cows.^15^ Drawing on scientific publications, institutional reports and archival materials dating from its initial identification in 1946, to c.1980 when current scientific understandings began to take hold, I will explore veterinary and scientific efforts to make sense of it through the drawing, redrawing and layering of disease identities.^16^ Examination of these processes not only illuminates the history of BVD, but also other conditions that it was variously associated with, to reveal how and why, in the wake of antibiotics, patterns and understandings of bovine disease changed.

BVD investigations were largely conducted in veterinary research contexts, where viruses of non-human animals were investigated apart from (though with awareness of developments in) both human medical virology and studies of viruses as biological phenomena. Their history sheds new light on these veterinary contexts, which do not feature in existing histories of virology.^17^ As the epicentre of BVD research, North America will form the focus of analysis, with the UK as an important point of comparison and connection. Events in Australia and Canada are also mentioned. While other Western European countries also encountered BVD, a detailed examination of their scientists’ perspectives falls beyond the scope of this article. To aid clarity, I will use the current label of ‘BVD’ throughout, while also indicating and interrogating the various names that scientists appended to it.

Like many viral diseases of the post-war period, there was nothing particularly distinctive about the symptoms of BVD. The first section will therefore address the circumstances and scientific methods that led to its identification and labelling as a disease. The second section explores how, over the next 25 years, developments in animal husbandry and virus research led to the discovery, in particular production systems, of additional BVD symptoms and viruses associated with them. These findings challenged initial observations about BVD, and brought new clinical, virological and epidemiological identities into being. The remaining two sections examine further shifts in the conceptualisation of BVD that resulted from efforts to control it by vaccination, and from newly identified associations with swine fever in pigs and Border Disease in sheep. These developments meant that by 1980, what had been a simple diarrhoeal disease of cows had been transformed into a highly complicated, variously presenting, multi-species phenomenon.

## Identification

In 1946, vets attached to the New York State Veterinary College (NYS-VC) reported the appearance of an ‘apparently new transmissible disease of cattle’ that they had identified in several herds of varying composition. Typical symptoms were: profuse diarrhoea, fever, lassitude, weight loss, ulcers in the mouth, muzzle and oesophagus, salivation, nasal discharge, inflamed mucous membranes, coughing and reduced milk production. Abortion and death were also reported.^18^ One of the report’s authors, FH Fox, was clinician to the Veterinary College’s ambulatory clinic. Later, he recollected being alerted to the disease when a cow he had diagnosed with ‘winter dysentery’ died unexpectedly. Another six herds that he went on to visit also developed symptoms, suggesting an infectious aetiology. This was confirmed by the other two authors, working in the Department of Pathology and Bacteriology, who succeeded in transmitting it to experimental cows via drenches of faeces and injections of blood from infected animals. Unable to isolate a causal bacterium, they deduced from the depressed white blood cell count that a virus was probably involved. These experiences led them to name the disease ‘Virus Diarrhoea’ (VD).^19^

It is worth reflecting on the circumstances that led them to identify ‘Virus Diarrhoea’ as ‘apparently new’. The emergence, spread and visibility of livestock infectious diseases were increasing at the time due to publicly funded efforts to ramp up agricultural production in a hungry post-war world. Encouraged by financial incentives and new approaches to livestock housing, husbandry and disease control, many farmers were growing their herds and increasing stocking densities. This encouraged the emergence and spread of infection. While the growing use of antibiotics removed many bacterial diseases from the picture, it had little effect on viral diseases, whose effects became noticeable. Vets gained more opportunities to encounter and investigate these diseases due to the publicly funded expansion of veterinary training, research and diagnostic services, and the emergence of a more affluent and educated farming population that was more inclined to seek veterinary aid.^20^

Typically, when presented with a case or outbreak of disease, veterinary (and medical) clinicians reached a diagnosis by comparing the signs, symptoms, history and population-level distribution to those of other, similarly presenting diseases. Focussing on those characteristics that appeared peculiar to the disease in question, and which enabled it to be differentiated from similar conditions, they weighed the competing diagnoses, selected the most likely, and based a course of action upon it. If this did not work, or if the disease’s presentation evolved over time, they reconsidered the situation, potentially drawing on routine post-mortems and laboratory tests to rule particular diagnoses in or out of the frame.^21^

This approach would not necessarily have led to the identification of BVD as a new disease. Its symptoms varied more in degree (namely duration and severity) than in kind from the condition known as ‘winter dysentery’, which was recorded in the UK as the third most frequent infectious disease of dairy cattle.^22^ Surviving records suggest that British government veterinary scientists overlooked BVD on at least one occasion when presenting symptoms pointed to no particular diagnosis, available tools did not permit visualisation of viral agents, and the farmer’s ‘bad management’ offered a ready catch-all explanation.^23^ So, what led vets at NYS-VC to pursue non-routine laboratory investigations that made apparent its viral aetiology? Their second publication provides a clue. It stated: ‘there were several things about this disease that made us wonder whether it was related to rinderpest’.^24^

Prior to its global eradication in 2011, rinderpest was the most contagious and fatal of all cattle diseases. Although the BVD mortality rate was much lower, its symptoms, lesions and infectious properties were not dissimilar. Rinderpest was largely confined to parts of Africa and Asia, but in Western countries during WWII, its use as a biological weapon had been widely feared. To prepare vaccine defences and (more covertly) explore offensive possibilities, a joint USA–Canada research programme had been located on the island of Grosse Isle, off the coast of Canada. This was scheduled for closure in 1947, but later revived when the onset of the Cold War provoked fears of biological attack by the Soviets.^25^

These circumstances prompted NYS-VC vets to investigate the relationship between BVD and rinderpest. Early results were not reassuring: two calves inoculated with serum from Grosse Isle that contained rinderpest antibodies did not fall ill when inoculated with material containing BVD virus. This suggested either that the diseases were similar, or that the serum contained antibodies to BVD as well as rinderpest.^26^ In a second experiment, they took cows that had recovered from BVD to Grosse Isle to be inoculated with rinderpest virus. Advised by James Baker, a graduate of NYS-VC who had participated in wartime rinderpest research, they discovered that the cows were vulnerable, meaning that the two diseases were distinct.^27^ Nevertheless, future calls for vigilance issued by veterinary commentators in the USA and Australia suggest that they were disturbed by the clinical resemblance of BVD to rinderpest. Fearing that practising vets might misdiagnose malicious introductions of rinderpest as BVD, they worked to raise the profession’s awareness of the resemblance between them, and of the need for laboratory diagnosis.^28^

Meanwhile, the US army (which had already intervened in wartime influenza research and vaccine development^29^) diverted funding to BVD research in the hope of better characterising the causal virus and developing a vaccine. This provided the new Veterinary Virus Research Institute at Cornell University with its first major grant. Supported also by private donations, the Institute formed part of a broader transition to the research university as the main site for biomedical research in the USA.^30^ It was founded in 1950 by virologist, James (better known as Drew) Baker, following the closure of the Rockefeller Institute’s Department of Animal and Plant Pathology at Princeton, where he had worked prior to his war-time secondment to rinderpest research. Like many other virus research settings in the early-mid twentieth century, the Rockfeller Institute had ranged widely across the viruses of humans, animals and plants. Subsequently, the dawning realisation that viruses were chemically and structurally distinct from bacteria led many virologists to study them as biological objects. By contrast, Baker’s Institute focussed specifically on viruses as agents of disease in dogs and cows. Taking its lead from wartime efforts to mobilise virology for the purpose of influenza and rinderpest vaccine production, it was to prove highly influential in the development of technologies to prevent animal viral diseases.^31^

In the UK, the first formal identification of BVD in 1953 was also precipitated by fears that a dangerous exotic virus had entered the country. This was not rinderpest but Malignant Catarrhal Fever (MCF). The subjects were a group of calves suffering from lack of appetite, nasal discharge, diarrhoea, coughing, laboured breathing, mouth ulcers, loss of condition and sporadic fatalities. The attending veterinary surgeon flagged the outbreak to the major publicly funded centre of applied veterinary research, the government’s Central Veterinary Laboratory (CVL) at Weybridge, Surrey.^32^ It launched an investigation, led by JH Hudson, Director of the newly created Virology Department. Hudson had spent over a decade working at the Central African Veterinary Research institute in Kenya, where he had gained considerable experience of MCF. Only after lengthy experimentation did he discount it as a potential diagnosis.^33^ Three years later in Kent, an outbreak of rinderpest was reported, prompting a flurry of visits from veterinary experts who also considered MCF as a possibility. Livestock movements were halted while they conducted further investigations, including inoculations of rinderpest serum, whose failure to protect cows from the disease dispelled alarm.^34^ CVL scientists grouped these disease incidents, and a handful of others they had been investigating, into a loosely defined entity that they believed to resemble that recently identified in the USA.^35^

## Clinical, Viral and Epidemiological Identities

The initial identification of BVD as a distinctive disease therefore resulted from laboratory investigations, which were prioritised and resourced on account of its clinical resemblance to exotic diseases that threatened national security. Subsequently, reports received from across North America and Europe broadened its identity beyond that of a rinderpest or MCF-like disease. These reports emanated mostly from veterinary colleges. They resulted from the combined efforts of clinicians, who reported on disease symptoms and distribution in the herd, and scientists, who investigated disease pathology, microbiology and attempted to reproduce it experimentally.

In 1953, vets from Iowa State University College of Veterinary Medicine encountered what they believed to be a condition similar to, but distinct from the ‘Virus Diarrhoea’ reported in NYS-VC. Although it, too, was characterised by ulcers in the mouth and gastrointestinal tract, it occurred more sporadically, and was more frequently fatal. They named it ‘Mucosal Disease’ (MD).^36^ Other reports from the USA, Australia and parts of Europe described clinical variants on this and Virus Diarrhoea. Vets debated whether they were the same or different entities, caused by one or more viruses.^37^ Additional insights emerged from the Glasgow University Veterinary College in 1956, when C Dow, Bill Jarrett and Ian McIntyre issued the first UK publication on ‘a disease…resembling the MD-VD complex’. They reported both overt clinical cases on which farmers had sought their advice, and cows with very mild symptoms, which they had discovered accidentally during field trials of a new parasitic lungworm vaccine.^38^

In revealing that the effects of BVD could range from frequently fatal to barely noticeable, these findings generated a much longer list of differential diagnoses. By 1957 it included a host of viral diseases (rinderpest, malignant catarrh, infectious rhinotracheitis, ulcerative stomatitis, ‘dirty mouth’, foot and mouth disease, infectious keratitis, ‘shipping fever’), bacterial diseases (salmonella, Johne’s disease), worm infestations, coccidiosis, dietary problems (vitamin A deficiency, acorn poisoning, corrosive poisoning) and the mysterious ‘X disease’ (later traced to the consumption of a manufactured wax used in certain lubricants).^39^ Speaking, in 1959, to a conference of Australian vets who were struggling to distinguish these conditions, KG Johnston from Sydney University stated that ‘The crying need in this whole problem is a detailed study of the viruses causing the various diseases recognised.’^40^

The characterisation of these viruses had been hampered by the fact that few could be visualised or cultured in vitro. Standard techniques for detecting their presence included passage through filters that retained bacteria, and inoculations of infective material into live cows. However, the relationships between them could only be assessed by testing whether the immunity generated by exposure to one virus protected cows against infection by another. Testing was slow, expensive and relied on a ready supply of susceptible cows (which Baker at Cornell happened to possess, having taken ownership of the disease-free herd that Dr Theobald Smith had created at the Rockefeller Institute, Princeton).^41^ It was complicated by the discovery that experimental transmission of the virus thought to be responsible for Mucosal Disease produced symptoms that were much milder than the naturally occurring disease.^42^

This type of mismatch between clinical experiences of disease and the outcome of laboratory-based efforts to characterise the virus believed responsible for it, had featured previously in US/UK investigations into influenza during the late 1930s and 1940s.^43^ Such difficulties were partly overcome by technical developments during the late 1950s. These made it easier to identify and characterise viruses. They also helped to confirm their status as a unique category of infectious agents, and to propel the establishment of virology as a field that was methodologically and institutionally distinct from bacteriology.

In addition to the ultracentrifuge and electron microscope, the plaque technique, developed in 1952 by Renato Dulbecco at the California Institute of Technology, was particularly important.^44^ It relied on the propensity of certain viruses (known as cytopathic, CP) to destroy host cells. On a newly created form of tissue culture made from a single layer of a single cell type, with antibiotics employed to depress bacterial contaminants, virus grew to produce visible plaques, whose numbers approximated to the quantity of virus present.^45^

The plaque technique was imported into British veterinary research contexts by RF Sellers, a virologist at the Foot and Mouth Disease research station, Pirbright, who visited Dulbecco at Caltech around 1954.^46^ At Florida University’s Department of Veterinary Science, AJ Kniazeff and WR Pritchard (who had reported the first appearance of a VD-like illness in Indiana in 1956) used it to investigate ‘Oregon C24V’. This was a CP strain of BVD isolated by James Gillespie at Cornell, which eventually became the prototype BVD virus used in labs across the world.^47^ Working to unravel the relationships between this and other viruses associated with BVD outbreaks in USA, UK and West Germany, they demonstrated in 1961 that a single type of virus was responsible.^48^ By 1967, although it was still not possible to reproduce Mucosal Disease symptoms experimentally, scientific opinion had coalesced around viewing this agent as responsible for both this and Virus Diarrhoea. Consequently, the disease became known as ‘BVD–MD’.^49^

While the emerging specialism of virology generated a singular viral identity for BVD–MD, it prompted further questioning of what veterinarians thought they knew about its clinical features. Like their colleagues in human medicine, who identified over 100 new viruses in the 1950s alone,^50^ virologists working in veterinary research contexts like Cornell or the UK CVL used their new tools to isolate and characterise a host of previously unrecognised viruses.^51^ They discovered that while in the laboratory, BVD could be attributed to a single virus type, in the field it presented frequently in combination with other viruses like Infectious Bovine Rhinotracheitis (IBR), papular stomatitis virus, myxovirus parainfluenza 3 (PI3) and adenovirus, whose respective contributions to presenting signs and symptoms were difficult to unravel.^52^

Similar findings were emerging in relation to other animal and human viral infections. Scientists had identified more viruses than there were diseases associated with them, and Koch’s longstanding postulates were of little help in distinguishing the viruses responsible for certain clinical syndromes.^53^ It seemed that ‘infection is not either a single entity or a fixed pattern. The picture, even of a single disease, is constantly liable to change surprisingly’.^54^ This made it harder than ever for clinicians to make sense of the symptoms they were witnessing.^55^ At the 1967 Australian Veterinary Association Congress, Professor DC Blood of Melbourne University complained that ‘there is no area of clinical medicine in which there is more difficulty in making a firm diagnosis than in the group of alimentary tract diseases of cattle characterised by lesions in the mouth and in which diarrhoea may or may not occur’.^56^ Laboratory aids were developed to address this situation: a gel precipitation diffusion test to identify rising antibody titres that indicated recent infection, and a Fluorescent Antibody test to rapidly identify the presence of virus within the body.^57^ However, neither were well suited for application in the field, where practitioners used patterns of signs and symptoms to guide their diagnoses, prognoses and therapeutic interventions, rather than turning immediately to laboratory tests.^58^

In human medicine, newly identified viruses were increasingly implicated as the major causes of acute respiratory illnesses, including bronchitis, which was one of the commonest conditions seen by general medical practitioners.^59^ Similar developments occurred in veterinary medicine. A combination of agents, including BVD, came to be associated with respiratory disease, especially in intensive systems of calf production where large numbers of animals were brought together at young ages from different destinations and under highly stressful conditions. Several weeks after their arrival, a significant proportion fell ill with fever, depression, a loss of appetite, reddening of the nasal membranes, ocular–nasal discharge, coughing, breathing difficulties and diarrhoea, which killed some calves and checked the growth of others. This condition was regarded as one of the costliest diseases to affect the cattle industry. Its features were reflected in its various names: ‘shipping fever’, ‘feedlot disease’, ‘virus pneumonia’, ‘calf pneumo-enteritis’, ‘enzootic pneumonia of calves’ (which was favoured in the UK) and ‘bovine respiratory complex’ (which was popular in the USA).^60^

The prevalence of respiratory disease was linked to changes in calf production. In North America, where new hybrid grains and irrigation techniques were generating more abundant harvests, findings from research into feeding all-grain diets to beef cattle encouraged owners of grain farms to create feedlots. Often supported by automated feeding technologies, these fattened large numbers of cattle from 6–7 months through to slaughter. Since the vast majority of breeding herds contained less than 50 cows, calves had to be purchased from many different sources, usually via auction markets. This involved lengthy stressful journeys, often without food and water, in which calves were exposed to many unfamiliar pathogens.^61^ Smaller-scale intensive systems were developing in the UK at the time, as expanded arable acreages, better crop fertilisers and higher yielding varieties of barley pushed output up and prices down. The concurrent development of larger, dedicated dairy farms generated a surplus of Friesian calves that could not be reared on site, leading to their pooling in specialist units. Some units reared calves to 3–6 months of age; others took them through to slaughter as 18-month-old ‘barley beef’. Both proved vulnerable to disease.^62^

Laboratory testing implicated various microbes in this condition, including BVD, infectious bovine rhinotracheitis (IBR), myxovirus parainfluenza 3 (PI3) and Pasteurella bacteria. According to veterinary scientific commentators, there was no point in trying to differentiate between their clinical effects, as they likely resulted from synergies between multiple disease agents. They saw antibody testing as an unreliable diagnostic tool because antibodies could be found in many animals that had no history of respiratory disease. They concluded that the respiratory condition should be treated as a complex rather than a group of clinically and aetiologically distinctive diseases.^63^ As a participant in the complex, BVD was awarded an additional clinical identity as a respiratory disease, and a new epidemiological identity as a feature of intensive calf systems. These operated in addition to its existing identity as a viral disease responsible for a range of diarrhoeal and mucosal symptoms.^64^

## Control and Complexity

Efforts to control BVD were motivated by fears of malicious rinderpest introduction, and the desire to avoid costly losses to dairy and beef production. The methods selected were shaped by what was known about its clinical, aetiological and epidemiological features. Its high prevalence, as revealed by large-scale serological testing, suggested there was little prospect of avoiding infection. As a viral disease, it could not be cured with antibiotics. Although the health implications of feedlot practices were widely acknowledged, they could not be abandoned easily due to the profits they realised and the capital investment they had required. Consequently, control efforts hinged on vaccination. Its anticipated benefits were informed by certain assumptions that scientists made about many infectious diseases at the time: that symptoms occurred when the singular viral agent of disease spread from infected to susceptible bodies, and that infection stimulated a life-long immune response.^65^

Vaccine production was led by Baker at Cornell, who boasted links between his Veterinary Virus Institute and ‘nearly every new vaccine produced in this country as well as many produced elsewhere for the prevention of disease in mammals other than man’.^66^ He employed live virus, which stimulated a stronger antibody response than killed virus, but removed its disease-causing properties by attenuation. The tendency of BVD to occur in combination with other viruses was addressed by incorporating some of them within the vaccine, making it cheaper to administer than multiple single-disease vaccines.^67^

Strong connections with local veterinarians and livestock producers underpinned the Institute’s field trials. These tested the practicality of combination vaccines in New York State milk herds, and used observations of vaccinated and unvaccinated stock in the same herd to evaluate vaccine effectiveness in preventing symptoms.^68^ The optimal timing of vaccination, antibody responses to it, duration of immunity and incidence of post-vaccinal reactions were subsequently investigated by the Dow Chemical Company, under laboratory conditions and in commercial feedlots. Controls were used in all studies. In 1964, Dow began to market a BVD vaccine and a combination BVD–IBR vaccine. By 1966, 3 million doses of each were produced annually in the USA, and a vaccine combining BVD, IBR, PI3 and leptospirosis was under development.^69^ Use of these vaccines was not permitted in the UK, where it was feared that modified live virus vaccines could revert to virulence and spread disease.^70^

To encourage the take-up of vaccination in US dairy herds, Baker, his PhD student RF Kahrs, and DS Robson from the Cornell Biometrics Unit, created a mathematical model. This used the known prevalence of BVD in the population, and the rate at which older animals (which were assumed to possess immunity through exposure to infection) were replaced by susceptible ones, to predict the likelihood of an outbreak.^71^ Further developed by Cornell statistician, Howard M Taylor, the model was used to inform the development of a vaccination schedule that balanced the cost of vaccination against the costs and risks of an epidemic outbreak.^72^ This was one of the first analyses of optimal resource allocation in disease control. Although its underpinning epidemiological assumptions were subsequently exploded, it is still cited today as an important influence on the mathematical modelling of infectious diseases, a method that was just emerging in the 1960s.^73^

Vaccines proved extremely popular in feedlots, where they were incorporated increasingly into ‘preconditioning programmes’. These recognised the additional role played by stress in precipitating the Bovine Respiratory Complex of which BVD formed a part. The first state-wide programme was developed in Iowa—which had more feedlots than any other US state—by John B Herrick, Extension Veterinarian at Iowa State University. Participating breeders not only vaccinated but also dewormed their calves, and weaned them onto a grain diet before sale. Purchasers were charged a premium for the health protection this provided. The scheme grew increasingly popular with producers and won endorsement from influential veterinary bodies. Other mid-Western states such as Illinois, Missouri, Ohio, Kentucky, Texas, South Dakota and Indiana followed suit.^74^

Vaccination did reduce the incidence of BVD in its various clinical forms. However, by the late 1960s, questions were being raised about its safety and efficacy. Reports of so-called post-vaccinal syndrome were increasingly received from feedlots. This resembled the mucosal form of BVD and ended frequently in death, 10–14 days after vaccination. In 1967, the American Veterinary Medical Association convened a symposium to discuss Bovine Respiratory Complex and the optimal methods for its control. Speakers from the Dow Company were quick to highlight the low incidence of post-vaccinal syndrome, the extensive testing that vaccines had undergone, and the lack of direct evidence implicating them. They suggested that the cows were to blame: they might have contracted BVD prior to vaccination, or their immune mechanisms might have failed, resulting in a lack of antibody production.^75^

While other participants challenged Dow’s attempt to make light of the problem,^76^ there was an emerging consensus that BVD epidemiology was more complex than originally thought. Just as in 1947, when the failure of previously successful influenza vaccines drove scientists to reconsider what they thought they knew about the causal virus,^77^ so experience of BVD vaccination prompted new questions. How did the BVD virus transmit and how did the body react to it? Was there a carrier state? Did infection result in immunity? How did management stresses and synergistic interactions between viruses impact on bovine physiology and pathology?^78^ Similar questions were being asked across the field of human and animal infectious disease, as scientific research and practical experience revealed that ‘we know far less about the common disease producing germs than many of us think we know’. They suggested the need for ‘an intelligent assessment of the best and most recent information available about the identity and the habits of disease-producing bacteria and viruses; about the state of immunity of the population; and about the effects of changes in our habits and social organization.’^79^

In the UK, where the only vaccines licensed for use were combination vaccines made from killed virus, post-vaccinal syndrome did not occur. However, studies on farms conducted in 1974 and 1979 showed that these vaccines had no significant effects on antibody levels or rates of respiratory disease.^80^ A follow-up study that isolated viruses and measured antibody levels in both healthy and sick calves indicated that as in children, respiratory virus infections were common and often asymptomatic. While BVD was found more frequently in calves with a higher incidence of respiratory disease, its incidence during outbreaks was not significantly higher than at other times. This led the authors to propose a new, more complex role for BVD in the causation of respiratory disease: rather than inducing symptoms in itself, it might prevent the bovine body from mounting an effective immune response to other respiratory viruses.^81^

## Dismantling Species Barriers

Running alongside these changes in the clinical, virological and epidemiological framings of BVD, were a series of investigations that challenged its identity as a bovine disease. Efforts to control BVD coincided with attempts to eradicate the highly infectious and extremely costly pig disease known as swine fever (hog cholera in North America), whose symptoms ranged from sudden death, to acute systemic disease, to chronic fever, inappetence and occasional abortion. Like BVD in feedlots, losses from swine fever mounted with the increased adoption of larger, more intensive husbandry systems. Unlike BVD, it was judged a threat to national livestock economies, and subjected to publicly funded eradication campaigns which proceeded by slaughtering diseased pigs and those exposed to infection. This approach was complicated by the variability of symptoms, which made it difficult to identify affected pigs. Although diagnostic tests were developed during the 1950s, they proved insensitive during the early stages, and could not be used in live pigs.^82^

In 1960, JH Darbyshire, a virologist at the UK government’s CVL, was charged with investigating whether infected pigs could be identified in life by means of a gel diffusion precipitin test, which bonded their serum antibodies to swine fever virus. He was already investigating this test as a method of BVD diagnosis. On applying it simultaneously to both viruses, he discovered—somewhat unexpectedly since the diseases appeared to bear little clinical resemblance to each other—that their antibody–virus complexes precipitated out at the same place in the agar gel, while those of another 10 viruses did not. BVD antibody bonded with swine fever virus, and vice versa, suggesting that the diseases had similar if not identical viral agents. Other forms of serological testing subsequently confirmed this finding, generating an opportunity for Darbyshire to tour the USA and network with its leading virologists who were working on BVD and swine fever.^83^

Baker and colleagues at Cornell attempted to apply this discovery to the control of disease in the field by using BVD vaccines to protect pigs from swine fever. They termed this approach ‘heterotypic vaccination’—as distinct from the standard ‘homotypic’ practice of preparing vaccines from the disease they were intended to prevent—and predicted its application across a range of diseases. They concluded that the protection offered by BVD vaccines was equivalent to that produced by swine fever vaccines, and that it was safer, because BVD virus could not spread between pigs. Research indicated that (for unknown reasons) the reverse did not apply: swine fever vaccination of cows did not protect them against BVD.^84^ Findings were sufficiently encouraging for a division of the Richardson–Merrell drug company to initiate the production and testing of BVD vaccines in pigs. However, results published in 1963 revealed inconsistent levels of protection.^85^

Meanwhile, Darbyshire’s findings took on new epidemiological significance in New South Wales, Australia, where the gel diffusion precipitin test was used to identify subclinical cases of swine fever. In 1962, positive results were obtained in a number of herds where no virus could be found.^86^ This prompted the question of whether the pigs had contracted BVD and developed antibodies that were cross-reacting with the test.^87^ Initially, there was no evidence that BVD infection of pigs could happen naturally in the field, but in 1973, researchers at the USDA’s National Animal Disease Laboratory in Iowa succeeded in isolating BVD virus from a sow and her piglets, and demonstrating its ability to reproduce and persist for several weeks. This revealed that while BVD virus might exhibit a preference for cows, it could also infect and be spread by pigs. It ruled out of court the prospect of using BVD vaccines in pigs for fear that the virus might survive in their bodies and spread to cattle.^88^

During the 1970s, the notion of BVD as a disease of bovines was challenged further by research that suggested a relationship with Border Disease in sheep. Named after its initial discovery in 1959, in sheep found on each side of the Welsh border, Border Disease manifested in the birth of weak, hairy, trembling lambs that grew slowly and had a high death rate. Those that survived to adulthood produced affected lambs of their own.^89^ Although initially it was thought to be hereditary, researchers in Sydney demonstrated in 1972 (8 years after its first identification in Australia) that Border Disease was transmissible. The inoculation of affected lamb brain and spinal cord into pregnant sheep caused some to abort and others to give birth to so-called 'hairy shakers'. Unexpectedly, the sheep that remained healthy were shown to have developed antibodies against BVD.^90^ Viewed in the light of an Australia-wide survey which showed that BVD antibodies were possessed by 5 to 10 per cent of sheep, it suggested an unspecified relationship between the Border Disease and BVD viruses.^91^

In a flurry of activity, researchers in the USA, UK, Ireland and Australia demonstrated that sheep could contract BVD through exposure to infected cows, that experimental infection of sheep with BVD produced clinical outcomes similar to Border Disease, and that cows were susceptible to the experimental transmission of Border Disease.^92^ Some lambs affected with Border Disease were shown to possess antibodies to swine fever, and in the gel diffusion precipitin test, all three viruses cross-reacted with each other’s antibodies.^93^ These findings prompted new questions about the relationships between disease agents that had been regarded as species specific: did the agent of swine fever cause Border Disease? Did the agent of BVD cause Border Disease? Or was there only one virus that transmitted across species? And what did this imply for disease control? Was it risky to pasture cattle and sheep together, or to feed bovine offal to pigs?^94^

While UK research suggested physical differences between Border Disease and BVD viruses,^95^ Australian researchers perceived a closer relationship, and spoke of a singular ‘Mucosal Disease virus’ of bovine or ovine origin.^96^ In 1975–76, when virus taxonomy was reorganised by the International Committee on Taxonomy of Viruses, Border Disease, BVD and swine fever were co-located in the new genus of pestiviruses.^97^ The organisation of livestock virus research tended to encourage the belief that they were distinctive, as institutions focussed on studying the disease that was of greatest significance to their local livestock economy (sheep in Scotland and Australia, pigs in the Netherlands and cattle in the USA) rather than the genus as a whole. Nevertheless, studies did cross-fertilise, and some researchers continued to question ‘whether it is justified to consider the pestiviruses... as distinct viral entities’.^98^

This cross-fertilisation was particularly important in enabling scientists to find answers to some of the questions thrown up by the failure of BVD vaccination in calves. It was already known that swine fever virus could cross the placenta, resulting in stillbirths or weak piglets that displayed congenital tremors, and in 1977, Dutch investigations revealed that some affected piglets survived for months and shed virus persistently.^99^ Meanwhile, at the Glenfield Veterinary Research Station, New South Wales, scientists reported the recovery of Border Disease virus from ‘hairy lambs’ but not normal ones, suggesting that they had experienced infection in the womb which had damaged or evaded their immune systems. Like swine fever-infected piglets, these lambs shed virus persistently. Some survived to adulthood and produced infected hairy lambs of their own.^100^

Subsequently, Cornell veterinary scientists demonstrated that BVD also behaved in this way.^101^ Experimental infections of cows during the early gestation period resulted in the birth of calves that remained persistently infected with BVD. One such calf survived well into its third year without ever developing BVD antibodies. This finding indicated why BVD vaccination sometimes failed. It also complicated understandings of how BVD spread,^102^ and inspired further investigations that revealed a host of additional clinical effects generated by the infection of pregnant cows. Early in gestation, abortion commonly resulted, while mid-term infections could result in calves with neurological defects. At a time when cash-strapped farmers were increasingly focussing on bovine reproductive performance as the key determinant of profit, these findings elevated the profile of BVD as ‘possibly the most important viral pathogen of the bovine fetus in Britain’.^103^ Along with the discovery, several years later, that the fatal form of Mucosal Disease occurred only in persistently infected and antibody-deficient cows that had been exposed to BVD in the womb, these insights transformed BVD into the reproductive, immunosuppressive, vertically spreading disease that it is still regarded as today.^104^

## Conclusion

In the 35 years following its initial identification, BVD was experienced as a complex and indeterminate disease whose identity was continually evolving. This study has revealed the influence exerted by changing agricultural practices, food production priorities, concerns about biological warfare, the technical and institutional evolution of virology, experiences of BVD in the field, the discovery of unexpected links with other diseases, and the unanticipated outcomes of vaccination. These influences produced multiple clinical, virological and epidemiological framings of BVD. In tracing the history of these framings, and the processes through which they were drawn, redrawn and superimposed, this article has revealed the impact of endemic BVD on bovine health, and illuminated the development of virology as applied to veterinary medicine.

By 1980, the label originally awarded to the disease—‘viral diarrhoea’—no longer made sense. Although initially identified in cows, it was now known that pigs and sheep could contract and spread it. The original defining symptom—diarrhoea—had been marginalised by its respiratory and reproductive effects, whose severity ranged from inapparent to fatal. While a singular viral agent had been identified, early ideas about its behaviours, effects and manner of control had been thrown into disarray. Its contribution to the Bovine Respiratory Complex was uncertain. Its relationships to swine fever and Border Disease were unclear. The discovery that it could cross the placenta had complicated understandings of transmission, while recognition of its immunosuppressive properties indicated why vaccination had proven less effective and more dangerous than originally anticipated.

Many health problems other than BVD have featured in this account of its history: the raft of other diarrhoeal and mucosal diseases from which vets sought to differentiate its symptoms; the various other respiratory viruses that were found to be present in the bodies it inhabited; and the diseases of other livestock species that shared its characteristics, and both complicated and contributed to its understanding and control. They feature partly on account of its biological tendency (shared by many other viruses) to occur in association with other infectious agents, resulting in a composite clinical picture whose relationship with infection was far from clear. These other viruses were also part of the process through which experts sought to make sense of BVD, by comparing and contrasting its clinical, microbial and epidemiological characteristics with those of other conditions that they knew more about, like rinderpest and swine fever.

Therefore, although at first sight, BVD may appear an obscure and unappealing historical subject, it actually offers a very powerful entrée into the demographics, experiences and understandings of endemic disease in post-WWII livestock farming. Arguably, it was the indeterminacy of BVD that encouraged scientists to identify its biological and conceptual associations with other diseases. Had it been easier to define, its relations would have been clearer and narrower, resulting in a more bounded account of its history.

Certain features of BVD set it apart from the many other viral diseases of humans and animals that rose to prominence in the post-WWII era. Fears of biological warfare account for the considerable attention and funding that were initially directed to its investigation, while its tendency to cross species boundaries made its epidemiology particularly difficult to unravel. Nevertheless, many contemporaneous endemic viral diseases were similarly indeterminate and challenging to identify and control. Therefore, BVD usefully exemplifies the sizeable set of poorly defined clinical conditions and loosely associated viral agents, whose definition, relationships and management preoccupied medical and veterinary scientists in the post-WWII decades.

Further historical case studies are now needed, of the likes of adenovirus, respiratory syncytial virus and enterovirus, and the respiratory and gastro-intestinal syndromes that were loosely associated with them. Such studies would usefully flesh out the kinds of disease concepts, scientific methodologies, specialist institutions and international communities that developed in association with these conditions at a time when their relative importance to health was increasing in Western countries. Such investigations into the prevalence, impacts and historical significance of diseases that were always present in some shape or form, would also provide an important counterbalance to a literature dominated by exceptional epidemic events. It would help to generate a more balanced and empirically grounded picture of the sorts of diseases that posed an ongoing challenge to human and animal bodies, the health professions, and the biomedical sciences in the antibiotic era.

## Funding 

This work was funded by the Wellcome Trust [grant number 209818/Z/17/Z]. For the purpose of open access, the author has applied a CC BY public copyright licence to any Author Accepted Manuscript version arising from this submission.

## Footnotes

1 Jeffrey Kahn and Kenneth McIntosh, ‘History and Recent Advances in Coronavirus Discovery’, *The Pediatric Infectious Disease Journal*, 2005, 24, S223–27; Nicola Decaro and Alessio Lorusso, ‘Novel Human Coronavirus (SARS-CoV-2): A Lesson from Animal Coronaviruses’, *Veterinary Microbiology*, 2020, 144, 108693.

2 Thomas Abraham, *Twenty-first Century Plague: The Story of SARS* (London: Johns Hopkins University Press, 2005); S. Harris Ali and Roger Keil, eds, *Networked**Disease: Emerging**Infections in the**Global**City* (Oxford: Wiley-Blackwell, 2008); Mark Honigsbaum, *The Pandemic Century: A History of Contagion from the Spanish Flu to Covid-19* (Cambridge: Penguin, 2020). On the human–animal intersections that characterise SARS, MERS and Covid-19, see Anita Guerrini, ‘Animals, Vaccines, and COVID-19’, *Endeavour*, 2021, 45, https://doi.org/10.1016/j.endeavour.2021.100779.

3 With the notable exception of Beth Greenhough, ‘Where Species Meet and Mingle: Endemic Human-Virus Relations, Embodied Communication and More-than-human Agency at the Common Cold Unit 1946–90’, *Cultural Geographies*, 2012, 19, 281–301.

4 Abigail Woods *et al.*, *One Health and**Its Histories: Animals and the Shaping of Modern Medicine* (Basingstoke: Palgrave, 2017).

5 Charles Rosenberg, ‘What Is an Epidemic? AIDS in Historical Perspective’, *Daedalus*, 1989, 188, 1–17, 2.

6
*Ibid*., 1.

7 Angela Creager and Jean-Paul Gaudillière, 'Experimental Arrangements and Technologies of Visualization: Cancer as a Viral Epidemic, 1930–1960’, in Jean-Paul Gaudillière and Ilana Löwy, eds, *Heredity and**Infection: The History of Disease Transmission* (London: Routledge, 2001), 203–41, 205. See also: Sally Smith Hughes, *The Virus: A History of the Concept* (London: Heinemann Educational Publishers, 1977); Ton van Helvoort, ‘History of Virus Research in the Twentieth Century: The Problem of Conceptual Continuity’, *History of Science*, 1994, 32, 185–235; P. Methot, ‘Writing the History of Virology in the Twentieth Century: Discovery, Disciplines, and Conceptual Change’, *Studies in the History and Philosophy of the Biological and Biomedical Sciences*, 2016, 59, 145–53.

8 For example: Virginia Berridge, *AIDS in the UK: The Making of a Policy, 1981-1994* (Oxford: Oxford University Press, 1996); Tamara Giles-Vernick and Susan Craddock, eds, *Influenza and Public Health: Learning from Past Pandemics* (London: Earthscan, 2010); Richard McKay, *Patient Zero and the Making of the AIDS Epidemic* (Chicago: University of Chicago Press, 2017); Lukas Engelmann, *Mapping AIDS: Visual Histories of an Enduring Epidemic* (Cambridge: Cambridge University Press, 2018); Rae-Ellen Kavey and Allison Kavey, *Viral Epidemics: From Influenza to Ebola and COVID-19* (London: Routledge, 2020); Honigsbaum, *Pandemic Century*.

9 Ulrike Lindner and Stuart Blume, ‘Vaccine Innovation and Adoption: Polio Vaccines in the UK, the Netherlands and West Germany, 1955–1965’, *Medical History*, 2006, 50, 425–46; Stephen Mawdsley, *Selling Science: Polio and the Promise of Gamma Globulin* (New Brunswick: Rutgers University Press, 2016); Gareth Millward, *Vaccinating Britain: Mass Vaccination and the Public since the Second World War* (Manchester: Manchester University Press, 2019).

10 Abigail Woods, *A Manufactured Plague: The History of Foot and Mouth Disease in Britain* (London: Earthscan, 2004); Peter Bartrip, *Myxomatosis: A History of Pest Control and the Rabbit* (London: Palgrave, 2008); A. McVety, *The Rinderpest Campaigns: A Virus, Its Vaccines, and Global Development in the Twentieth Century* (Cambridge: Cambridge University Press, 2018). The role of viruses in the causation of cancer has also attracted considerable attention, for example Creager and Gaudillière 'Experimental Arrangements’.

11 Maurice Hilleman *et al*., ‘Acute Respiratory Illnesses Among Children and Adults’, *Journal of the American Medical Association*, 1962, 180, 445–53; John T. Macfarlane and Michael Worboys, ‘The Changing Management of Acute Bronchitis in Britain, 1940–1970: The Impact of Antibiotics’, *Medical**History*, 2008, 52, 47–72.

12 Alfred Evans, ‘Causation and Disease: The Henle-Koch Postulates Revisited’, *The Yale Journal of Biology and Medicine*, 1976, 49, 175–95.

13 Michael Thrusfield, *Veterinary Epidemiology* (London: Butterworths, 1986); Abigail Woods, ‘Decentring Antibiotics: UK Responses to the Diseases of Intensive Pig Production, c1925-65’, *Palgrave Communications*, 2019, 5, 41.

14 Ilana Löwy, ‘Epidemiology, Immunology, and Yellow Fever: The Rockefeller Foundation in Brazil, 1923-1939’, *Journal of the History of Biology*, 1997, 30, 397–417; Creager and Gaudillière, 'Experimental Arrangements’; Leo Slater, *War and Disease: Biomedical Research on Malaria in the Twentieth Century* (London: Rutgers University Press, 2009).

15 V. Richter *et al*., ‘A Systematic Worldwide Review of the Direct Monetary Losses due to Bovine Viral Diarrhea Virus Infection’, *Veterinary Journal*, 2017, 220, 80–87. 

16 The only existing histories of BVD are by scientists and/or actors, and adopt an internalist perspective. For example: Anon, ed, *International Symposium: Bovine Viral Diarrhea Virus, a 50 Year Review* (Cornell University: The College of Veterinary Medicine, 1996); Denise Goens, ‘The Evolution of Bovine Viral Diarrhea: A Review’, *The Canadian Veterinary Journal*, 2002, 43, 946–54; Dirk Deregt, ‘Introduction and History’, in Sagar Goyal and Julia Ridpath, eds, *Bovine Viral Diarrhea Virus: Diagnosis, Management and Control* (Ames: Blackwell, 2005), 3–33.

17 Historians of virology have tended to highlight the lack of boundaries between research into human and animal viruses. However, such accounts rarely extend beyond the 1950s, and are often more concerned with the study of viruses as biological phenomena than with veterinary and medical efforts to control them. for example, Hughes, *The Virus*; Lisa Wilkinson and AP Waterson, *An**Introduction to the**History of**Virology* (Cambridge: Cambridge University Press, 1978); Daniel Kevles, ‘Renato Dulbecco and the New Animal Virology: Medicine, Methods, and Molecules’, *Journal of the History of Biology*, 1993, 26, 409–42; Angela Creager, *The Life of a Virus: Tobacco Mosaic Virus as an Experimental Model, 1930-65* (London: University of Chicago Press, 2002). There are no dedicated histories of virology as applied to veterinary medicine.

18 P. Olafson, A. D. MacCallum and F. H. Fox, ‘An Apparently New Transmissible Disease of Cattle’, *Cornell Veterinarian*, 1946, 1, 205–13.

19 F. H. Fox, ‘Historic Clinical Perspective’, in Anon, ed, *International Symposium*, 1–4.

20 Abigail Woods, ‘Is Prevention Better Than Cure? The Rise and Fall of Veterinary Preventive Medicine, c1950-80’, *Social History of Medicine*, 2013, 26, 113–31; Woods, ‘Decentring Antibiotics’.

21 J. Done, ‘The Use of Pathology in the Control of Disease in Farm Livestock’, *State Veterinary Journal*, 1964, 19, 102–8.

22 F. W. Withers, E. F. Peck and F. B. Leech, ‘A Survey of Diseases and Losses in Dairy Cattle in Wiltshire and Devonshire’, *Veterinary Record*, 1959, 572–80.

23 Correspondence from G. B. S. Heath to D. W. Menzies, 9 December 1953, National Archives, MAF 189/603.

24 P. Olafson and C. G. Rickard, ‘Further Observations on the Virus Diarrhea (New Transmissible Disease) of Cattle’, *Cornell Veterinarian*, 1947, 37, 104–06, 105.

25 McVety, *The Rinderpest Campaigns.* The WWII programme was run by Richard Shope, a virus expert who had discovered the cause of swine influenza. He was previously based at the Department of Animal Pathology, Rockefeller Institute for Medical Research, Princeton.

26 Olafson and Rickard, ‘Further Observations’.

27 Olafson, MacCallum and Fox, ‘An Apparently New Transmissible Disease’; R. V. L. Walker and P. Olafson, ‘Failure of Virus Diarrhea of Cattle to Immunize against Rinderpest’, *Cornell Veterinarian*, 1947, 37, 107–11.

28 I. A. Schipper *et al*, ‘Mucosal Disease of Cattle’, *Veterinary**Medicine*, 1956, 50, 431–34; K. G. Johnston, ‘The Virus Diarrhoeas of Cattle and Similar Diseases’, *Australian Veterinary Journal*, 1959, 35, 101–10.

29 Jean-Paul Gaudilliere, ‘Rockefeller Strategies for Scientific Medicine: Molecular Machines, Viruses and Vaccines’, *Studies in**History**and**Philosophy of**Biology**& Biomedical**Sciences*, 2000, 31, 491–509.

30 Memorial Statement for Professor James Andrew Baker, 1975. https://ecommons.cornell.edu/handle/1813/18142 (last accessed 13 January 2021); James Gillespie, ‘Cornell’s Contributions to BVD MD Research’, in Anon, ed, *International Symposium*, 5–10; Creager, *The Life of a Virus*.

31 The Institute Report from the Veterinary Virus Research Institute (1952), https://ecommons.cornell.edu/handle/1813/31310/recent-submissions?offset=60 (last accessed 13 January 2022); Hughes, *The Virus*; Wilkinson and Waterson, *An Introduction*; Kevles, ‘Renato Dulbecco’; van Helvoort, ‘History of Virus Research’; Gaudilliere, ‘Rockefeller Strategies’; Creager, *The Life of a Virus*; John Eyler, ‘De Kruif’s Boast: Vaccine Trials and the Construction of a Virus’, *Bulletin of the**History of**Medicine*, 2006, 80, 409–38; McVety, *The Rinderpest Campaigns*.

32 Anon, *Animal Health, a Centenary* (London: HMSO, 1965), 311–25.

33 J. R. Hudson and A. Steele Bodger correspondence 1953, NA MAF 189/603.

34 General correspondence, March–June 1956, NA MAF 189/855.

35 L. M. Markson correspondence, June 1956, NA MAF 189/855.

36 F. K. Ramsey and W. H. Chivers, ‘Mucosal Disease of Cattle’, *North American Veterinarian*, 1953, 34, 629–33.

37 H. Hedstrom and A. Isaksson, ‘Epizootic Enteritis in Cattle in Sweden’, *Cornell Veterinarian*, 1951, 41, 251–53; Ramsey and Chivers, ‘Mucosal Disease’; S. W. Nielsen *et al*., ‘Mucosal Disease of Cattle in Ontario’, *Canadian Journal of Comparative Medicine and Veterinary Science*, 1955, 19, 318–24; C. Dow, W. F. H. Jarrett and W. I. M. McIntyre, ‘A Disease of Cattle in Britain Resembling the Virus Diarrhoea Mucosal Disease Complex’, *Veterinary**Record*, 1956, 70, 620–23; Schipper *et al*., ‘Mucosal Disease’; D. C. Blood *et al*., ‘Mucosal Disease of Cattle in Australia’, *Australian Veterinary Journal*, 1957, 33, 75–76.

38 Dow, Jarrett and McIntyre, ‘A Disease of Cattle’.

39 Olafson, MacCallum and Fox, ‘An Apparently New Transmissible Disease’; Dow, Jarrett, and McIntyre, ‘A Disease of Cattle’; Schipper *et al*., ‘Mucosal Disease’; United States Department of Agriculture, *Hyperkeratosis (X-Disease) of Cattle* (Leaflet no. 355, Washington, D.C, 1954). R. A. Huck, ‘Mucosal Disease Complex’, *Journal of Comparative Pathology and Therapeutics*, 1957, 67, 267–76.

40 Johnston, ‘Virus Diarrhoeas of Cattle’, 108.

41
*The Institute Report from the Veterinary Virus Research Institute*, 1960, 10, 6.

42 W. R. Pritchard, ‘The Bovine Viral Diarrhea-Mucosal Disease Complex’, in C. A. Brandley and E. L. Jungherr, eds, *Advances in Veterinary Science* (New York: Academic Press, 1963), 1–47.

43 Eyler, ‘De Kruif’s Boast’.

44 Hughes, *The Virus*; van Helvoort, ‘History of Virus Research’; N. Rasmussen, *Picture Control. The Electron Microscope and the Transformation of Biology in America, 1940-1960* (Stanford: Stanford University Press, 1997); Creager and Gaudillière, ‘Experimental Arrangements’; Creager, *The Life of a Virus*.

45 Kevles, ‘Renato Dulbecco’.

46 Obituary, R. F. Sellers, *Veterinary**Record*, 2011, 169, 130–31.

47 J. H. Gillespie, J. A. Baker and K. Mcentee, ‘A Cytopathogenic Strain of Virus Diarrhea Virus’, *Cornell Veterinary*, 1960, 1, 73–79.

48 A. J. Kniazeff and W. R. Pritchard, ‘Antigenic Relationships in the Bovine Viral Diarrhea-Mucosal Disease Complex’, *Proceedings US Livestock Sanitary Association*, 1961, 344–50; A. J. Kniazeff *et al*., ‘Antigenic Relationship of Some BVD Viruses from the United States, Great Britain and West Germany’, *Veterinary**Record*, 1961, 73, 768–69.

49 Gillespie, ‘Cornell’s Contributions’.

50 A. S. Evans, ‘Common Clinical Syndromes in Infectious Disease’, *Wisconsin Medical Journal*, 1960, 59, 508–12, 508.

51 During the early 1960s, the CVL focussed its virology resources on isolating and classifying as many cytopathic viruses as they could from outbreaks of BVD, respiratory disease and viral infertility. R. A. Huck Correspondence, 1960, NA MAF 224/235.

52 Correspondence, ‘Virus Stomatitis in Calves’, 1956–62, NA MAF 189/854; A. B. Paterson, ‘Virus Diseases in Calves’, *Veterinary**Record*, 1962, 74, 1384–94; J. H. Darbyshire, ‘The Isolation, Separation and Identification of Two Viruses from a Case of Bovine Mucosal Disease’, *Journal of**Comparative**Pathology and**Therapeutics*, 1963, 73, 309–20; R. A. Huck and Sheila Cartwright, ‘Isolation and Classification of Viruses from Cattle During Outbreaks of Mucosal or Respiratory Disease and from Herds with Reproductive Disorders’, *Journal of**Comparative**Pathology and**Therapeutics*, 1964, 346–65.

53 R. J. Huebner, ‘The Virologist’s Dilemma’, *Annals of the New York Academy of Sciences*, 1957, 67, 430–38.

54 J. W. Howie, ‘Infectious Disease - Does It Still Matter?’, *Public Health*, 1968, 82, 253–68, 254.

55 C. J. York, ‘Differential Diagnosis of Bovine Virus Diseases’, *Proceedings US Livestock Sanitary Association*, 1961, 485–92.

56 D. C. Blood, ‘Field Procedures for the Differential Diagnosis of Mucosal Disease in Cattle’, *Australian**Veterinary**Journal*, 1967, 43, 501–05.

57 J. H. Darbyshire, ‘Agar Gel Diffusion Studies with a Mucosal Disease of Cattle: I.—Preliminary Results with the Technique’, *Research in Veterinary Science*, 1962, 3, 118–24.

58 F. K. Ramsey *et al*., ‘Field Cases of Bovine Respiratory Disease in Iowa’, *Journal of**American**Veterinary**Medical**Association*, 1968, 152, 751–56, 755.

59 Macfarlane and Worboys, ‘Acute Bronchitis in Britain’.

60 R. Jensen and D. Mackey, *Diseases of Feedlot Cattle* (Philadelphia: Lea & Febiger, 1965); J. F. Harbourne, ‘Survey of Bovine Respiratory Diseases with Special Reference to the Serological Examination of Paired Serum Samples’, *Veterinary**Record*, 1966, 78, 749–52; D. G. McKerchner, ‘Bovine Respiratory Infections’, *Journal of**American**Veterinary**Medical**Association*, 1968, 152, 729–38; R. Pierson, ‘Control and Management of Respiratory Diseases in Beef Cattle’, *Journal of American Veterinary Medical Association*, 1968, 152, 920–23; J. I. H. Phillip, ‘Viral Respiratory Diseases’, *Veterinary**Record*, 1968, 82, clinical supplement no. 14, iv–vi.

61 D. T. Ensley, ‘Attitudes and Experiences with the Iowa Beef Cattle Preconditioning Program: A Survey of Feedlot Operators’ (Master of Science dissertation, Iowa State University, 2001).

62 A. K. Smith, ‘The Healthy Calf: A Guide to Rearing and Management to Reduce Mortality’, *Farmers Weekly*, 2 October 1964, 80–90; Harbourne, ‘Survey of Bovine Respiratory Diseases’; M. McG Cooper and M. B. Willis, *Profitable Beef Production* (Ipswich: Farming Press Ltd, 1972), 127–37.

63 McKerchner, ‘Bovine Respiratory Infections’; Ramsey *et al.*, ‘Field Cases of Bovine Respiratory Disease’.

64 L. B. Fastier, ‘The Role of Vaccination in the Control of Bovine Respiratory Virus Disease’, *New Zealand Veterinary Journal*, 1969, 17, 117–25.

65 By 1966, 52% of cows in New York State had been exposed to BVD. R. F. Kahrs, D. S. Robson and J. A. Baker, ‘Epidemiological Considerations for the Control of Bovine Virus Diarrhea’, *Proceedings US Livestock Sanitary Association*, 1966, 145–53.

66
*The Institute Report from the Veterinary Virus Research Institute*, 1960, 10, 19.

67
*Ibid*.; *The Institute Report from the Veterinary Virus Research Institute*, 1957, 7; James Baker, Immunization of Cattle against Virus Diarrhea Virus, United States Patent, filed 1 February 1962, issued 20 December 1966. https://patents.google.com/patent/US3293129A/en (last accessed 13 January 2022).

68 Robert Kahrs, ‘Studies on Combination Vaccines for Dairy Cattle’ (unpublished PhD thesis, Cornell University, 1965).

69 J. M. Bittle, ‘Vaccination for Bovine Viral Diarrhea-Mucosal Disease’, *Journal of American Veterinary Medical Association*, 1968, 152, 861–68; J. M. Bittle and C. York, ‘Combination Vaccines for the Control of Bovine Respiratory Diseases’, *Journal of American Veterinary Medical Association*, 1968, 152, 889–893; J. Bittle, ‘Field Use of Bovine Vaccines’, *Journal of Dairy Science*, 1970, 53, 625–27. Bittle was the Head of the Department of Infectious Diseases at the Dow Chemical Company.

70 R. F. Gordon and J. S. Garside, ‘Newcastle Disease Vaccination’, *Veterinary**Record*, 1965, 76, 20–21; A. B. Paterson response to correspondence from F. G. Brown, 30 October 1963, NA MAF 189/922.

71 D. S. Robson, R. F. Kahrs and J. A. Baker, ‘Bounds on the Mean Recurrence Time’, *Journal of Theoretical Biology*, 1967, 17, 47–56.

72 H. Taylor, ‘Some Models in Epidemic Control’, *Mathematical Biosciences*, 1968, 3, 383–98.

73 B. Armbruster and M. Brandeau. ‘Cost-Effective Control of Chronic Viral Diseases: Finding the Optimal Level of Screening and Contact Tracing’, *Mathematical Biosciences*, 2010, 224, 35–42.

74 Ensley, ‘The Iowa Beef Cattle Preconditioning Program’. In 1968 there were 46,000 feedlots in Iowa, with the most rapid growth occurring in those with over 1,000 cattle. J. L. Anderson, *Industrialising the Corn Belt: Agriculture, Technology, and Environment, 1945-72* (DeKalb: Northern Illinois University Press, 2009).

75 P. Chennekatu, D. E. Tyler and F. K. Ramsey, ‘Characteristics of a Condition Following Vaccination with Bovine Virus Diarrhea Vaccine’, *Journal**of the**American Veterinary Medical Association*, 1967, 150, 46–52; S. F. Rosner, ‘Complications Following Vaccination of Cattle against IBR, BVD-MD, and PI3’, *Journal of American Veterinary Medical Association*, 1968, 152, 898–902.

76 L. N. Brown and F. K. Ramsey, ‘Comments on Complications Following Vaccination of Cattle against Bovine Respiratory Diseases’, *Journal of American Veterinary Medical Association*, 1968, 152, 903–04; C. V. Peacock, ‘Comments on Complications Following Vaccination of Cattle against Bovine Respiratory Diseases’, *Journal of American Veterinary Medical Association*, 1968, 152(6), 905–07.

77 Eyler, ‘De Kruif’s Boast’.

78 ‘Report of the Panel for the Symposium on Immunity to the Bovine Respiratory Disease Complex’, *Journal of American Veterinary Medical Association*, 1968, 152, 713–19.

79 J. W. Howie, ‘Infectious Disease - Does It Still Matter?’, *Public Health*, 1968, 82, 253–68, 254.

80 L. H. Thomas and A. P. Collins, ‘Virus Infection and Incidence of Respiratory Disease: A Serological Study of Four Micro-Organisms at a Large Beef-Rearing Farm’, *Veterinary**Record*, 1974, 94, 506–09; S. P. Morzaria *et al*., ‘A Field Trial with a Multicomponent Inactivated Respiratory Viral Vaccine’, *Veterinary**Record*, 1979, 105, 410–14.

81 E. J. Stott *et al*., ‘A Survey of Virus Infections of the Respiratory Tract of Cattle and Their Association with Disease’, *Epidemiology & Infection*, 1980, 85, 257–70.

82 P. R. Ellis, *An Economic Evaluation of the Swine Fever Eradication Programme in Great Britain*, Study no. 11 (University of Reading, Department of Agriculture, 1972); Ole Stalhaim, ‘The Hog Cholera Battle and Veterinary Professionalism’, *Agricultural History*, 1988, 62, 116–21. Swine fever was eradicated from Australia in 1963, the UK in 1966 and the USA in 1977.

83 J. H. Darbyshire, ‘Agar Gel Diffusion Studies with a Mucosal Disease of Cattle: II. A Serological Relationship between a Mucosal Disease and Swine Fever’, *Research in**Veterinary**Science*, 1962, 3, 125–28. Darbyshire was invited by Bill Pritchard, Head of the Veterinary Science Department of the University of Florida. He also visited the new US Department of Agriculture laboratory at Ames, Iowa, and the Veterinary Virus Research Institute at Cornell. J. Darbyshire, ‘A Visit to Florida and Other Parts of the United States’, *State Veterinary Journal*, 1962, 17, 239–43.

84 Baker and colleagues also attempted to protect dogs from distemper using a vaccine made from human measles virus, and from infectious hepatitis virus using human adenovirus. *The Institute Report from the Veterinary Virus Research Institute*, 1962, 12, 1; J. A. Baker *et al*., ‘Possibility of Hog Cholera Eradication with BVD Vaccine’, *Proceedings of the United States Livestock Sanitary Association*, 1963, 366–70.

85 Peter Langer, ‘Development of a Heterotypic Bovine Virus Diarrhea (BVD) Vaccine against Hog Cholera’, *Proceedings of the United States Livestock Sanitary Association*, 1963, 358–65.

86 The same discovery was made in the USA over a decade later, when its swine fever eradication programme neared completion. E. A. Carbrey *et al*., ‘Natural Infection of Pigs with Bovine Viral Diarrhea Virus and Its Differential Diagnosis from Hog Cholera’, *Journal of American Veterinary Medical Association*, 1976, 169, 1217–19. By contrast in Britain, no cross-reactions were detected. J. I. H. Phillips and J. H. Darbyshire, ‘Infection of Pigs with Bovine Viral Diarrhoea Virus’, *Journal of**Comparative**Pathology*, 1972, 82, 105–09.

87 J. C. Keast and N. K. Golding, ‘Further Developments in Relation to Swine Fever in New South Wales’, *Australian**Veterinary**Journal*, 1964, 40, 137–44; W. A. Snowdon and E. L. French, ‘The Bovine Mucosal Disease-Swine Fever Virus Complex in Pigs’, *Australian**Veterinary**Journal*, 1968, 44, 179–84.

88 A. L. Fernelius *et al*., ‘Bovine Viral Diarrhea Virus in Swine: Characteristics of Virus Recovered from Naturally and Experimentally Infected Swine’, *Canadian**Journal of**Comparative**Medicine*, 1973, 37, 13–20.

89 L. E. Hughes, G. F. Kershaw and I. G. Shaw, ‘“B” or Border Disease: An Undescribed Disease of Sheep’, *Veterinary**Record*, 1959, 71, 313–17.

90 Helen Acland, G. P. Gard and J. W. Plant, ‘Infection of Sheep with a Mucosal Disease Virus’, *Australian**Veterinary**Journal*, 1972, 48, 70.

91 T. D. St George, ‘A Survey of Sheep throughout Australia for Antibody to Parainfluenza Type 3 Virus and to Mucosal Disease Virus’, *Australian**. Veterinary**Journal*, 1971, 47, 370–74.

92 G. M. Ward, ‘Experimental Infection of Pregnant Sheep with Bovine Viral Diarrhea-Mucosa Disease Virus’, *Cornell Veterinary*, 1971, 61, 179–91; A. F. Hamilton and P. J. Timoney, ‘Bovine Virus Diarrhoea/Mucosal Disease Virus and Border Disease’, *Research in**Veterinary**Science*, 1973, 15, 265–67; E. L. French *et al*., ‘Infection of Pregnant Ewes with Mucosal Disease Virus of Ovine Origin’, *Australian**Veterinary**Journal*, 1974, 45–54; D. F. Gibbons *et al*., ‘Pathogenicity of the Border Disease Agent for the Bovine Foetus’, *British**Veterinary**Journal*, 1974, 130, 357–60; W. A. Snowdon, I. M. Parsonson and Mary Lou Broun, ‘The Reaction of Pregnant Ewes to Inoculation with Mucosal Disease Virus of Bovine Origin’, *Journal of**Comparative**Pathology*, 1975, 85, 241–51.

93 J. W. Plant *et al*., ‘Immunological Relationship between Border Disease, Mucosal Disease and Swine Fever’, *Veterinary**Records*, 1973, 455; Bennie Osburn *et al*., ‘Border Disease-like Syndrome in Lambs: Antibodies to Hog Cholera and Bovine Viral Diarrhea Viruses’, *Journal of American Veterinary Medical Association*, 1973, 163, 1165–67.

94 French *et al*., ‘Infection of Pregnant Ewes’.

95 J. T. Vantsis *et al*., ‘Experiments in Border Disease: VIII Propagation and Properties of a Cytopathic Virus’, *Journal of**Comparative**Pathology*, 1976, 86, 111–20.

96 French *et al*., ‘Infection of Pregnant Ewes’; W. A. Snowdon, I. M. Parsonson and Mary Lou Broun, ‘The Reaction of Pregnant Ewes to Inoculation with Mucosal Disease Virus of Bovine Origin’, *Journal of**Comparative**Pathology*, 1975, 85, 241–51.

97 International Committee on Taxonomy of Viruses, *Second Report*, 1976. https://talk.ictvonline.org/taxonomy/p/taxonomy_releases (last accessed 13 January 2022).

98 J. T. Van Oirschot, ‘Congenital Infections with Nonarbo Togaviruses’, *Veterinary Microbiology*, 1983, 8, 321–61.

99 J. T. Van Oirschot and C. Terpstra, ‘A Congenital Persistent Swine Fever Infection. I. Clinical and Virological Observations’, *Veterinary Microbiology*, 1977, 2, 121–32.

100 G. P. Gard, H. M. Acland and J. W. Plant, ‘A Mucosal Disease Virus as a Cause of Abortion, Hairy Birth Coat and Unthriftiness in Sheep’, *Australian**Veterinary**Journal*, 1976, 52, 64–68; J. W. Plant, G. P. Gard and Helen Acland, ‘Transmission of a Mucosal Disease Virus Infection Between Sheep’, *Australian**Veterinary**Journal*, 1977, 53, 574–77.

101 Oirschot, ‘Congenital Infections’.

102 G. M. Ward *et al*., ‘A Study of Experimentally Induced Bovine Viral Diarrhea-Mucosal Disease in Pregnant Cows and Their Progeny’, *Cornell Veterinary*, 1969, 59, 525–38; R. F. Kahrs, F. W. Scot and A. de Lahunta, ‘Bovine Viral Diarrhea-Mucosal Disease, Abortion, and Congenital Cerebellar Hypoplasia in a Dairy Herd’, *Journal of American Veterinary Medical Association*, 1970, 156, 851–57; F. W. Scott *et al*., ‘Virus Induced Congenital Anomalies of the Bovine Fetus. I. Cerebellar Degeneration (Hypoplasia), Ocular Lesions and Fetal Mummification Following Experimental Infection with Bovine Viral Diarrhea-Mucosal Disease Virus’, *Cornell Veterinary*, 1973, 63, 536–60; M. F. Coria and A. W. McClurkin, ‘Specific Immune Tolerance in an Apparently Healthy Bull Persistently Infected with Bovine Viral Diarrhea Virus’, *Journal of American Veterinary Medical Association*, 1978, 172, 449–51.

103 J. T. Done *et al*., ‘Bovine Virus Diarrhoea-Mucosal Disease Virus: Pathogenicity for the Fetal Calf Following Maternal Infection’, *Veterinary**Records*, 1980, 106, 473–79.

104 Deregt, ‘Introduction and History’.

## Notes

Abigail Woods is PVC and Head of the College of Arts at the University of Lincoln. Her research focusses on the history of animals, animal health and livestock agriculture in modern Britain.

